# Socio-demographic and social support factors related to substance use in South African in-school adolescents: Insights from the Girls Achieve Power (GAP Year) trial in three peri-urban settings

**DOI:** 10.12688/gatesopenres.13422.1

**Published:** 2021-11-09

**Authors:** Kerry Gordon, Alison Kutywayo, Sasha Frade, Nicolette Naidoo, Saiqa Mullick

**Affiliations:** 1 Wits Reproductive Health & HIV Institute (Wits RHI), University of the Witwatersrand, Johannesburg, 2193, South Africa; 2 Demography and Population Studies Programme, University of the Witwatersrand, Johannesburg, 2000, South Africa

**Keywords:** substance use, social support, adolescent, mental health, South Africa, GAP Year

## Abstract

**Background: **Substance use is a significant public health problem worldwide, with consequences including violence, risky behaviours, and even death. Substance use amongst adolescents is increasing in South Africa, and limited research on frequency, risk and protective factors means that prevention interventions are difficult to design. This paper aims to describe and discuss factors associated with substance use among school-going adolescents in three peri-urban South African settings.

**Methods: **A cross-sectional analysis was conducted using baseline data from participants in the Girls Achieve Power (GAP Year) trial. Grade 8 learners (N=2383), aged 11-18, were recruited from 26 lowest quintile public high schools in three townships: Soweto and Thembisa in Gauteng Province, and Khayelitsha in Western Cape Province. A baseline survey gathered demographic and behavioural data. Questions relevant to substance use and social support were used for this analysis. Multivariate logistic regression analyses were conducted to identify factors associated with substance use. The final variables were included in an unadjusted and adjusted logistic regression for current substance use, and a multinomial logistic regression for frequency of substance use.

**Results:** A total of 22.5% (534) of participants indicated they had ever used substances. Being male was strongly associated with substance use (P<0.001), and less strongly with frequency of substance use. Age significantly predicted substance use, with older adolescents being more likely to engage in substance use (P<0.001); having a parent/guardian employed was negatively associated with substance use (P=0.021). Family-related social support variables were predictive of substance use. Being able to count on friends when things went wrong was predictive of lower frequency of substance use (P=0.019).

**Conclusions:** These results can inform the targeting of prevention interventions to males and younger learners, as well as ensuring youth interventions build family and peer support to make substance use less likely and less frequent.

## Introduction

Substance use is defined as taking a substance into the body for an intended purpose, such as behavioural or emotional change ( Marshall & Spencer, 2018). The term ‘substance’ can include a range of legal and illegal substances such as alcohol and drugs (e.g., cigarettes, marijuana, heroin, cocaine, prescription medication not used as prescribed, inhalants such as paint thinners). Substance use is a public health concern, as it has been found to be a predictor of injury, violence, risky sexual behaviour, and a risk factor for HIV and TB acquisition ( Flisher *et al.*, 2012; Morojele & Ramsoomar, 2016). In 2016, alcohol alone was responsible for 3 million deaths and 132.6 million disability-adjusted life years (DALYs) worldwide ( WHO, 2018). Use of substances may occur without having a significant impact on an individual’s functioning, but when tolerance is built and use increases, even at the expense of work or school, relationships, or one’s own health or safety, this can indicate a substance use disorder. Substance use disorders are characterised by a growing addiction or dependence on the substance, and an inability to curb one’s use ( American Psychiatric Association (APA), 2013). These disorders are associated with various mental health and psychosocial problems and have the potential to reinforce broader socio-economic inequalities ( World Health Organisation (WHO), 2018).

In South Africa (SA), substance use, particularly alcohol use, is common, increasingly among adolescents ( Dada *et al.*, 2019). However, national-level data on substance use is not collected regularly, and there is thus a lack of recent information about levels of use, and risk factors for use have received limited attention ( Morojele & Ramsoomar, 2016). School surveys in settings such as Johannesburg ( Mohale & Mokwena, 2020), Cape Town ( Morojele *et al.*, 2011) and East London ( Manu & Maluleke, 2017) have found alcohol to be the most commonly used substance, followed by cannabis and cigarettes. In rural areas, prevalence of substance use may be lower than in urban samples ( Tshitangano & Tosin, 2016). Studies conducted in SA have found several demographic factors to be associated with adolescent substance use, including being male, age (evidence is mixed on the direction of the relationship), friends using substances, having lower income, or a parent being unemployed ( Brook *et al.*, 2006; Magidson *et al.*, 2017; Mohale & Mokwena, 2020; Muchiri & dos Santos, 2018; Peltzer *et al.*, 2010). Internationally, longitudinal studies have found substance use to be associated with male gender, younger age, and the presence of common mental health disorders such as depression and anxiety ( Brook *et al.*, 2006; Lai *et al.*, 2015).

Compounding the problem is the fact that treatment options for substance use disorders in SA are limited, and even more so for adolescents ( Dada *et al.*, 2019). Substance use is most likely to be initiated during adolescence ( United Nations Office on Drugs and Crime (UNODC), 2018), partly due to the nature of that developmental stage, which is often associated with increased impulsivity and risk-taking, intensified emotional arousal, and a high value placed on peer influence ( Magidson *et al.*, 2017; Petersen *et al.*, 2012). Adolescents are thus an important target group for substance use prevention interventions.

The causes of substance use disorders are numerous and complex. Some factors which may make substance use more likely to lead to substance abuse and addiction are a genetic or neurological vulnerability to addiction, poor impulse control, mental health problems (such as anxiety, depression or post-traumatic stress disorder), peer pressure, lack of parental supervision, access to the substance, low social support, and less access to resources ( UNODC, 2018). Social support has been shown to be a protective or at least a moderating factor for substance use in a few studies, but the source and nature of support make a difference, with parental support having a more positive influence than peer support ( Measelle *et al.*, 2006; Muchiri & dos Santos, 2018; Oetting & Donnermeyer, 1998; Oxford *et al.*, 2001). 

Contributing to the limited body of literature on substance use in adolescents, this paper aims to describe and discuss factors which predict substance use and the frequency of substance use among in school-going adolescent participants in the Girls Achieve Power (GAP Year) trial, using baseline data. GAP Year is a cluster randomised controlled trial (cRCT), testing the effectiveness of a four-pronged ecological intervention: a sports-based after-school asset-building intervention with content aligned to the national curriculum on sexuality education, a parent intervention, linkage to care and school safety which includes the implementation of the NSSF ( Kutywayo *et al.*, 2018). The primary outcomes are to reduce school dropout of adolescent girls between grades 8–10 and increase reporting of gender-based violence (GBV).

## Methods

### Research Questions

This paper uses baseline data from the GAP Year trial to address the following research questions:

1. How many participants have ever used substances, how many are currently using substances, and which substances have they used?
2. Which socio-demographic and social support variables are associated with current substance use (in the last year) among participants?
3. Which socio-demographic and social support variables are associated with the frequency of substance use among participants?

### Study Design and Setting

A baseline cross sectional survey was conducted in 3432 Grade 8 participants enrolled into the GAP Year cRCT, between April 2017 – September 2018. This paper presents the analysis and findings of a secondary data analysis of the main study baseline data.

Participants completed a knowledge, attitudes, perceptions, and behaviour (KAPB) survey at baseline. Twenty-six low-quintile ^i^ public high schools in three townships were selected in Soweto and Thembisa Townships in Gauteng Province (GP) and Khayelitsha Township in Western Cape Province (WC), SA. Site selection was done using data to assess the high burden of HIV, GBV and pregnancy. Schools were selected using the following criteria: mixed sex public high schools in Thembisa, Soweto and Khayelitsha; in quintiles 1–3; which had not been exposed to any asset building interventions in the past six months. A one to one (1:1) stratified randomization scheme was used to assign the 26 schools to intervention or control groups (13 in each).

### Study Participants and Sample Size

All Grade 8 learners at selected schools were eligible to participate in the baseline survey, irrespective of age, race, or sex. The grade 8 learner age range is approximately 12–14 years; however, due to learners repeating grades and other reasons, the age range is often wider with older learners enrolled up to 18 years ( Barnes *et al.*, 2012; Branson *et al.*, 2014).

### Measures

The baseline interview consisted of an interviewer-administered questionnaire collecting information on demographics, socio-economic status and knowledge and attitudes pertaining to school safety, social support and social networks, sexuality, gender and norms, sexual reproductive health and rights (SRHR) and care-seeking behaviours. Following completion of this component of the interview, the participant then completed a behavioural audio computer-assisted self-interview (ACASI), allowing learners to hear questions through headphones and respond on a tablet themselves, aiming to reduce social desirability bias. The ACASI section, 20–30 minutes in duration, asked sensitive questions regarding the participant’s actual practices and behaviour, covering questions on socio-economic status, social support and social networks, sexual and reproductive health, GBV, and substance use. The majority completed the interviewer-administered survey first, although this was not always the case, depending on learner availability.

For this paper, questions about current (in the last year) and ever use of alcohol, smoking, legal and illegal drugs were analysed. Participants were asked about the frequency of use, and this data is included in this analysis. It should be noted that due to the structure of the questionnaire, individual participants’ data on substance use and frequency was not linked to particular substances, or number of substances, they used.

### Data Management and Analysis

Data from the baseline interviews was collected and stored using REDCap (Research Electronic Data Capture) digital data capture tools hosted by the University of Witwatersrand ( Harris *et al.*, 2009; Harris *et al.*, 2019). REDCap is a secure, web-based software platform which provides 1) an easy-to-use interface for capturing validated information; 2) audit trails which track any data manipulation and export processes; 3) automated export processes allowing data downloads to most statistical packages; and 4) procedures for data integration and interoperability with external sources. Survey data was stored on encrypted password-protected tablets and the synced data was stored on secure servers by Wits RHI. Data was exported from REDCap and ACASI systems, and subsequently into StataCorp 2017 ( StataCorp, 2017).

Bivariate percentage and frequency tables were used to show the distribution of the population by the selected socio-demographic and social support factors, by substance use and frequency of substance use. Pearson Chi² test for association was calculated for the selected outcomes and factors. These analyses were done to ascertain the level of association between the selected independent variables and substance use and frequency of use, respectively. Only those statistically significant at a significance level of 0.05 were retained in the regression models.

Regression analyses were conducted on the data to ascertain the relationships between demographic variables (sex, age, residing with parent/guardian, parent/guardian’s employment status and whether parent/guardian was receiving a grant), as well as mental health and social support variables (e.g., “I can talk about my problems with my friends”), with participant substance use and the frequency of substance use. The final and selected variables were included in an unadjusted and adjusted logistic regression for substance use, and a multinomial logistic regression for frequency of substance use. Odds ratios and relative risk ratios were calculated, and P-values are all interpreted at a 0.05 level of significance. It should be noted that the “No” category has been removed for dichotomous variables [variables with a Yes or No Response] shown in the tables in the Results, except for participant substance use.

### Ethical approval and considerations

Ethical approval for the study was received from the University of the Witwatersrand Human Research Ethics Community (#M160940). The Western Cape Department of Education (WCDE) and Gauteng Department of Education (GDE) provided Provincial research approval. Learners’ enrolment and participation were voluntary, and they were free to refuse to respond to any questions. Only those with written parental/guardian consent and individual written assent were recruited into the study. Study social workers were available to provide counselling interventions and linkage to further care to participants during data collection. Social harm forms and procedures were developed for effective referrals to psychosocial support, where required.

All data collection was supervised by the research team. Initially in English, the questionnaires were translated into isiXhosa as this language is commonly spoken at some study sites. Interview environments were designed to ensure participant confidentiality. Where feasible, interviewers were the same sex as the participant and participants were free to stop the interview process at any time. 

## Results

### Socio-Demographic and Substance Use Variables

Overall, 3432 eligible learners participated in the baseline survey. We included 2383 in the analysis as this was the number who completed both the behavioural and ACASI surveys. Table 1 provides a summary of the socio-demographic variables of these participants. The majority (63.1%, n=1504) were female, and most (81.4%, n=1938) were aged between 12-14 years. The mean age of participants was 13.7 years with a standard deviation of 0.95. Forty one percent (n=967) of the participants resided with both parents, 40.9% (n=954) with a single parent, and 17.7% (n=414) with a relative or guardian. Over two-thirds of the participants (68.8%, n=1633) reported that at least one of their parents/guardians was employed. A similar proportion (67.3%, n=1498) had a parent/guardian who received a social grant. Socio-demographic variables are summarised in Table 1.

**Table 1.** Summary of socio-demographic variables of participants (n=2383).

	N (%)
**Sex**	
Female	1 504 (63.1)
Male	879 (36.9)
**Age**	
12–14 Years	1 938 (81.4)
15–18 Years	443 (18.6)
*Missing*	*2*
Mean [SD)	13.7 [0.95]
**Reside with**	
Both parents	967 (41.4)
Single parents	954 (40.9)
Relative/guardian	414 (17.7)
*Missing*	*48*
**Parent/Guardian Employment**	
At Least One Parent/Guardian Employed	1 633 (68.8)
*Missing*	*8*
**Social Grants**	
Parent/ Guardian Receives Grant	1 498 (67.3)
*Missing/don’t know*	*157*

‘Ever use’ of substances, including specific substances used, is summarised in Table 2. Twenty two percent of all participants (n=534) indicated that they had ever used substances, with the majority (86.1%, n=460) only using one substance. The most frequently used substances were alcohol (32.7%, n=223), and dagga (cannabis) (23.6%, n=161) followed by cigarettes (10.3%, n=70). Twelve participants did not answer the question on ‘ever use’ of substance. Almost 75.5% of ever users (n=403) had used in the last year (data not shown). Of current users, 40% (n=161) had used substances at least once a week, 24% (n=98) had used every few weeks, and 35.7% (n=144) had only ever used once or twice (data not shown).

**Table 2.** ‘Ever use’ of substances use among adolescents.

	% (N)
**Ever Used a Substance (n=2 371)**	
No	77.4 (1 837)
Yes	22.5 (534)
**Number of Substances Used (n=534)**	
1 Substance	86.1 (460)
2–3 Substances	11.2 (60)
4 or More Substances	2.6 (14)
**Substances Ever Used ^1^ (n=682)**	
Alcohol	32.7 (223)
Dagga ^2^	23.6 (161)
Cigarettes	10.3 (70)
Cocaine	9.1 (62)
Rohypnol	3.7 (25)
Glue	3.2 (22)
Mandrax ^3^	3.1 (21)
Nyaope ^4^	2.8 (19)
Ecstasy	1.9 (13)
Heroin	1.8 (12)
Downers ^5^	0.9 (6)
LSD	0.9 (6)
Crystal Meth	0.7 (5)
Speed	0.6 (4)
Special K ^6^	0.6 (4)
Other	4.3 (29)

^1^ Multiple responses allowed ^2^ Cannabis ^3^ A sedative medication usually crushed, mixed with cannabis and smoked ^4^ A street drug containing antiretroviral medication mixed with other substances, often heroin and cannabis ^5^ Nervous system depressants such as opiates, barbiturates, benzodiazepines and antihistamines ^6^ Slang term for ketamine

### Social Support Variables

All participants were asked about the social support they received from family and friends ( Table 3), as well as services available for social support. A minority of participants (4.5%, n=107) reported being aware of any mental health and psychosocial services being offered in local health facilities (data not shown).

**Table 3.** Participant responses to social support questions.

Social Support Variable	n	%
“My family really tries to help me” (N=2 374)	2 309	97.3
“I get the emotional help and support I need from my family” (N=2 374)	2 250	94.8
“I can talk about my problems with my parents/guardians” (N=2 369)	2 038	86.0
“My family is willing to help me make decisions” (N=2 369)	2 201	92.9
“My friends really try to help me” (N=2 369)	2 074	87.6
“I can count on my friends when things go wrong” (N=2 368)	1 759	74.3
“I can talk about my problems with my friends” (N=2 369)	1 800	76.0
“I have friends with whom I can share my joys and sorrows” (N=2 372)	2 108	88.9

In terms of social support from family, 97.3% (n=2309) of participants felt their family really tried to help them, and 94.8% (n=2250) reported receiving the emotional support they needed from their families. When asked about social support from friends, 87.6% (n=2074) of participants felt their friends really tried to help them, and 74.3% (n=1759) thought they could count on their friends when things went wrong.

Table 3 provides a summary of frequencies and proportions of participants’ responses to the social support variables under investigation. All social support variables had a small number of missing responses. 

### Substance Use

Table 4 shows the differences in socio-demographic and social support variables by current substance use. Just over a fifth of the 879 boys (22.4%, n=197) but a smaller proportion of the 1504 girls (13.7%, n=206) who responded to the questionnaire were currently using substances (P<0.001). About a quarter (24.2%, n=107) of the 443 participants in the older age group (age 15-18) were using substances, but only 15.3% of the 1938 12–14-year-olds (n=296) were current substance users (P<0.001).

**Table 4.** Socio-demographic and social support variables by substance use in the last year.

	Substance Use in the Last Year		P-Value
	Yes	No	
	% (N)	% (N)	
Sex (n=2 371)			*<0.001*
Female	51.1 (206)	65.4 (1 287)	
Male	48.9 (197)	34.6 (681)	
Age Category (n=2 369)			*<0.001*
12–14 Years	73.5 (296)	83.1 (1 633)	
15–18 Years	26.6 (107)	16.9 (333)	
Reside With (n=2 323)		*0.58*	
Both parents/guardian	39.3 (156)	41.8 (805)	
Single parents	41.6 (165)	40.7 (785)	
Relative/guardian	19.1 (76)	17.5 (336)	
Parent/Guardian Employed (n= 2363)	63.9 (257)	69.6 (1 365)	*0.03*
Parent/Guardian Receives Grant (n=2 216)	67.5 (255)	67.2 (1 235)	*0.92*
Aware of mental health and psychosocial services offered (n= 2371)	2.7 (11)	4.88 (96)	*0.06*
“My family really tries to help me” (n=2 362)	95.5 (383)	97.7 (1 916)	*0.01*
“I get the emotional help and support I need from my family” (n=2 362)	92.3 (371)	95.3 (1 867)	*0.02*
“I can talk about my problems with my parents/guardians” (n=2 357)	83.3 (334)	86.6 (1 693)	*0.09*
“My family is willing to help me make decisions” (n=2 357)	90.8 (364)	93.3 (1 825)	*0.07*
“My friends really try to help me” (n=2 357)	85.5 (342)	88.0 (1 723)	*0.16*
“I can count on my friends when things go wrong” (n=2 356)	74.4 (296)	74.4 (1 457)	*0.99*
“I can talk about my problems with my friends” (n=2 357)	74.8 (300)	76.3 (1 493)	*0.52*
“I have friends with whom I can share my joys and sorrows” (n=2 360)	87.5 (349)	89.2 (1 750)	*0.30*

A significant difference was observed based on participants’ parents’/guardians’ employment: 63.9% (n=257) of current substance users had at least one parent/guardian who was employed, and 69.6% (n=1365) of those not currently using had at least one parent or guardian employed (P=0.03).

A significantly higher percentage of those who were not using substances (97.7%, n=1916) compared to those who were using substances (95.5%, n=383) stated that their families really tried to help them when they were in need (P=0.01). Those not currently using substances (95.3%, n=1867) were significantly more likely than current substance users (92.3%, n=371) to report that they received the emotional support they needed from their families (P=0.02).

Only those factors shown to be statistically significant at the bivariate stage were included in the final regression model for substance use in the last year. The results for the unadjusted and adjusted models are shown in Table 5.

**Table 5.** Unadjusted and adjusted regression models for predictors of current substance use.

	Unadjusted Odds Ratio [CI] *P-Value*	Adjusted Odds Ratio [CI] *P-Value*
***Sex***		
Female	1 [1.0-1.0]	1 [1.0-1.0]
Male	1.81 [1.456-2.244] *<0.001*	1.77 [1.414-2.203] *<0.001*
***Age***		
12–14 Years	1 [1.0-1.0]	1 [1.0-1.0]
15–18 Years	1.77 [1.380-2.277] *<0.001*	1.62 [1.252-2.091] *<0.001*
***Parent/Guardian Employed***		
No	1 [1.0-1.0]	1 [1.0-1.0]
Yes	0.77 [0.618-0.970] *0.03*	0.76 [0.606-0.960] *0.02*
***“My family really tries to help me”***		
Agree	1 [1.0-1.0]	1 [1.0-1.0]
Disagree	1.68 [1.101-2.557] *0.02*	1.54 [0.998-2.391] *0.05*
***“I get the emotional help and support I need from my family”***		
Agree	1 [1.0-1.0]	1 [1.0-1.0]
Disagree	1.29 [0.964-1.731] *0.09*	1.29 [0.949-1.740] *0.11*

Unadjusted regression revealed that sex, age, parent/guardian employment and feeling their family really tried to help them were significantly associated with substance abuse in the last year. Males were 81% more likely to be currently using substances, compared to females (OR 1.81; 95% CI 1.45-2.24; P<0.001). Those aged 15–18 years were 77% more likely to be currently using a substance compared to those aged 12–14 years (OR 1.77; 95% CI 1.38 – 2.27; P<0.001). Not having at least one parent/guardian employed was a significant predictor of current substance use, with substance use in the last year being 23% less likely for those who had a parent/guardian employed compared to those who did not have a parent or guardian employed (OR 0.77; 95% CI 0.618-0.970; P=0.03). Current substance users were 1.68 times more likely to have disagreed with the statement “My family really tries to help me” compared to those who had not used substances (OR 1.68; 95% CI 1.101-2.557; P=0.02).

When adjusted, sex, age, and parent/guardian employment remained significant. Males were 77% more likely to be currently using substances compared to females (aOR 1.77; 95% CI 1.41 – 2.20; P<0.001). Those 15-18 years were 1.62 times more likely to be currently using a substance compared to those aged 12-14 years (aOR 1.62; 95% CI 1.252-2.091; P<0.001). Those who had at least one parent/guardian employed were 24% less likely to have used substance in the last year (aOR 0.76; 95% CI 0.606-0.960; P=0.02) than those who did not. The statement “My family really tries to help me” was marginally significant, with current substance users being 54% more likely to disagree with this statement than those not using substances (aOR 1.54; 95% CI 0.998-2.391; P=0.05).

### Frequency of Substance Use

Table 6 indicates how socio-demographic and social support variables differed according to the frequency of substance use. Frequency was grouped into the following three categories: At least once a week (frequent substance users), Every few weeks (occasional substance users), and Only once or twice (infrequent substance users).

**Table 6.** Demographic and social support variables of current substance users by frequency of substance use.

	Frequency of Substance Use [n=461]			*P-Value*
	At least once a week	Every few weeks	Only once or twice	
	% (N)	% (N)	% (N)	
Sex				
Female	43.9 (87)	46.4 (51)	64.7 (99)	*<0.001*
Male	56.1 (111)	53.6 (59)	35.3 (54)	
Age Categories
12–14 Years	69.2 (137)	78.2 (86)	75.8 (116)	*0.17*
15–18 Years	30.8 (61)	21.8 (24)	24.2 (37)	
Reside with
Both parents	38.8 (76)	38.5 (42)	40.00 (60)	*0.86*
Single parents	41.8 (82)	41.3 (45)	44.7 (67)	
Relative/ guardian	19.4 (38)	20.2 (22)	15.3 (23)	
*missing*	*(2)*	*(1)*	*(3)*	
Parent Employed	63.1 (125)	64.8 (70)	66.7 (102)	*0.79*
Parent Receives Grant	66.2 (123)	65.4 (66)	70.4 (100)	*0.63*
Aware of mental health and PSS services offered	2.0 (4)	2.7 (3)	4.6 (7)	*0.38*
“My family really tries to help me”	96.4 (189)	97.3 (107)	93.5 (143)	*0.26*
“I get the emotional help and support I need from my family”	91.9 (181)	96.4 (106)	91.5 (140)	*0.26*
“I can talk about my problems with my parents/guardians”	88.3 (174)	81.8 (90)	79.6 (121)	*0.07*
“My family is willing to help me make decisions”	89.9 (177)	90.8 (99)	92.8 (142)	*0.63*
“My friends really try to help me”	82,7 (162)	83.6 (92)	91.5 (139)	*0.05*
“I can count on my friends when things go wrong”	72.6 (143)	66.1 (72)	80.0 (120)	*0.04*
“I can talk about my problems with my friends”	71.6 (141)	76.4 (84)	76.3 (116)	*0.51*
“I have friends with whom I can share my joys and sorrows”	82.2 (162)	88.2 (97)	89.3 (134)	*0.13*

Frequency of substance use differed significantly for the following variables: sex, feeling their friends really try to help, and being able to count on their friends when things go wrong.

Table 6 shows the sex variation within each frequency. Only 5.8% (n=87) of the 1504 females in the trial reported using substances very frequently (i.e., at least once a week), whereas 12.6% (n=111) of the 879 males in the trial reported frequent substance use (P<0.001). Similarly, 3.4% (n=51) of all females reported using substances every few weeks, in comparison to 6.7% (n=59) of all males (P<0.001).

In terms of social support variables, most participants reported feeling supported by family and friends on all measures. Overall, family support appeared to be reported more frequently than support from friends. Only the following variables reached significance according to frequency of substance use:

- “My friends really try to help me” (P=0.05)
- “I can count on my friends when things go wrong” (P=0.04)

The likelihood of a participant being aware of psychosocial services in their community increased as the frequency of substance use decreased, although the differences were not significant.

Only the three factors shown to be statistically significant at the bivariate stage were included in the final regression model for substance use, with two showing significant differences. The results for the unadjusted model are shown in Table 7.

**Table 7.** Unadjusted regression analysis for frequency of substance use [RC: At least once per week].

	At Least Once Every Few Weeks	Only Once or Twice
	Relative Risk Ratio [CI] *P-Value*	Relative Risk Ratio [CI] *P-Value*
**Sex**		
Female	1 [1.0-1.0]	1 [1.0-1.0]
Male	0.91 [0.568-1.448] *0.68*	0.43 [0.279-0.660] *<0.001*
**“My friends really try to help me”**		
Agree	1 [1.0-1.0]	1 [1.0-1.0]
Disagree	1.36 [0.821-2.255] *0.23*	0.66 [0.398-1.100] *0.11*
**“I can count on my friends when things go wrong”**		
Agree	1 [1.0-1.0]	1 [1.0-1.0]
Disagree	0.93 [0.498-1.743] *0.83*	0.45 [0.226-0.878] *0.02*

The regression analysis indicated that, compared to frequent substance users (at least once per week), males were 57% less likely than females to be infrequent substance users (using only once or twice) (RRR 0.43; CI 0.279-0.660; P<0.001). Occasional substance use (at least once every few weeks) did not vary significantly by sex.

Only one indicator of social support was found to vary significantly by frequency of substance use. The least frequent substance users were 55% more likely than frequent substance users to feel that they could count on their friends when things went wrong (RRR 0.45; CI 0.226-0.878; P=0.02).

None of these variables remained significant in an adjusted regression analysis.

## Discussion and conclusions

The results give an overview of substance use activities amongst GAP Year participants, highlighting that 22.5% have ever used substances. Whilst the proportion of respondents who reported using substances is lower than has been found in some South African school-age samples (e.g. Mohale & Mokwena, 2020; Morojele *et al.*, 2011), about half of ever users went on to use more regularly. Many adolescents will only ever experiment with substances, and although experimentation cannot be altogether prevented, this also represents a pivotal time for intervention. Effective interventions can target the risk factors which make continued or habitual use more likely.

We found that alcohol was the most frequently used substance, followed by cannabis and cigarettes: this is similar to the breakdown of learner substance use surveyed in South African schools ( Manu & Maluleke, 2017; Mohale & Mokwena, 2020; Morojele *et al.*, 2011). This trend may reflect broader sociocultural patterns of high alcohol use in South Africa ( Popova *et al.*, 2017), which may make alcohol easier to access and more acceptable than other substances. Alcohol carries significant risks to brain development, mental health and school performance and retention ( World Health Organisation (WHO), 2018). Substance abuse interventions would thus have maximum impact if they targeted alcohol use – but this would also mean considering the complexities of South Africans’ relationship with alcohol.

Sex appears to be most strongly associated with substance use in this population, with males significantly more likely to engage in substance use, and use substances more frequently than females. This reflects substance use trends locally and worldwide: males are more likely to use substances than females, and females who do use tend to do so less than males ( Peltzer & Phaswana-Mafuya, 2018; WHO, 2018). Consistently over the past decade, an overwhelming proportion (over 80% across provinces) of South African adolescents receiving treatment for substance abuse are male ( Dada *et al.*, 2019). There are various reasons why substance use may be gendered. Males are generally found to be more risk-taking ( Reniers *et al.*, 2016), even from a neurological perspective ( Victor *et al.*, 2015), and social norms in many countries encourage this ( Mahalik *et al.*, 2015). Given that adolescence is already a period in which risk-taking and impulsiveness is increased, this places adolescent males at a markedly high risk of engaging in substance use. Interventions for substance use might benefit from being more tailored to gender-specific risk factors and motivators, as well as being targeted more strongly to males.

Age was also found to be a predictor of substance use (though not of frequency), with older participants more likely to engage in substance use, reinforcing findings from several South African studies (e.g. Magidson *et al.*, 2017; Mohale & Mokwena, 2020; Morojele *et al.*, 2011). It should be noted that older participants in this study are likely to have other characteristics which put them at higher risk, as they would all be at an advanced age for their school grade. The relationship with age could also suggest that perception of the risk of substance use decreases in the course of adolescence in this population. Although neurological research tends to suggest that risk-taking behaviour decreases with maturity ( Steinberg, 2008), some studies have found the opposite – that older adolescents may, in some contexts, be more likely to take risks, perhaps influenced by the freedoms and privileges they might attain as they get older ( Reniers *et al.*, 2016). Risk-taking has been estimated to peak around age 18 years in some studies ( Steinberg, 2008). This indicates that substance use primary prevention interventions would be most effective if targeting younger adolescents or even preadolescents, and that secondary or tertiary prevention of substance use can be targeted at older adolescents who are more likely to already be engaging in substance use. Interventions explored in research or practice tend to target populations who have already been identified as engaging in substance use ( Carney *et al.*, 2020), indicating a gap in primary prevention.

Socio-economic factors, such as who the adolescent resides with and whether they received grants, did not emerge as consistent predictors of substance use or its frequency, but there is evidence to suggest that a parent or guardian who is employed (and thus provides consistent financial support) could be a protective factor against substance use – and conversely that parent unemployment is a risk factor ( Muchiri & dos Santos, 2018). This reinforces theories that substance use may be fuelled by a need to escape harsh socio-economic conditions ( Peltzer *et al.*, 2010).

Perceived social support also seems to have some correlation with substance use, with one family-related measure of support emerging as predictive of substance use itself, and two friend-related indicators differing by frequency of use for those who had used substances in the last year. This confirms research findings that caregivers act as a moderator or mediator for other risk factors children and adolescents may be facing ( Measelle *et al.*, 2006; Petersen *et al.*, 2012), and that strong parental attachment relationships play a role in whether an adolescent uses substances ( Brook *et al.*, 2006). Although research exists on peer substance use influencing adolescents’ substance use, there is limited knowledge about peer support, especially in a South African context. Although these results are not conclusive, their significance indicates that social support from family may differ amongst current substance users compared to non-substance users. Thus, relationships and felt support from family and peers may play some role in the complex aetiology of substance use behaviours in this context. They also highlight the importance of including parents, guardians and peers in substance use prevention strategies, which often necessitates using a holistic or socio-ecological approach to interventions.

Although it was not a significant predictor of substance use or frequency, it should be noted that awareness of mental health and psychosocial support services is troublingly low in this population, with likelihood of knowing about services lowest amongst those who used substances most frequently, and thus need it most. This may reflect the reality that there are insufficient mental health services in South Africa relative to the needs of the population, especially for children and adolescents ( Docrat *et al.*, 2019). However, it is important for young people to be made aware of services that do exist, so that substance use or its causes can be treated as far as possible. In the long term, government, donors and community structures must recognise the central role of mental health in public health, as well as its clear comorbidities with pandemics such as HIV, TB and COVID-19 ( United Nations (UN), 2020).

Adolescent substance use presents both a mental health and public health challenge in South Africa, especially given its multiple pathways of causation and the limits to availability of treatment. Prevention of substance use is therefore the most effective strategy for reducing harm, and continuing research to guide policy and interventions for substance use in adolescents is crucial to protect this population from the destructive effects of substance use and abuse.

The complexity of the nature of substance use disorders makes it particularly difficult to design interventions to prevent or mitigate substance use, and often governments and healthcare providers are working under budget constraints, with small allocations (around 5%) being made for mental health ( Docrat *et al.*, 2019). Understanding which factors place adolescents at higher risk of substance use, and which factors can be protective, can allow for both prevention and treatment interventions to be appropriately designed and targeted.

### Strengths, Limitations and Recommendations for Future Research

When reviewing these findings, the following strengths and limitations should be considered. This study was conducted in 26 schools in three culturally and socio-economically diverse townships in South Africa, and thus the results have some generalisability. The high numbers of respondents for most questions allowed for valid statistical analyses to be conducted.

However, analysis of the baseline questionnaire was limited by variations in the number of respondents between questions, meaning that true numbers were not necessarily reflected in these statistics. This could be addressed by structuring questionnaires to ensure participants answer all questions. The inconsistencies in the numbers of participants who reported using substances between questions in the questionnaire suggests an uncertainty, lack of understanding, or discomfort about disclosing substance abuse. Some questions aimed to elicit potentially sensitive or socially unacceptable responses (e.g., whether a participant uses substances), meaning that social desirability bias may have influenced responses. This reflects a general challenge in administering self-report measures regarding substance use, especially with adolescents. Further efforts could be made to ensure the reduction of social desirability bias. Research on adolescent substance use could also use alternative or indirect ways of questioning participants to gain a true sense of adolescents’ behaviours.

Future studies could structure questionnaires to ask in more depth about substance use (e.g., specific symptoms of addiction) as well as a wider range of mental health variables, as the association between substance use and mental health symptoms is relatively unexplored in the South African adolescent population ( Magidson *et al.*, 2017). In addition to family support, the influence of peers is a potentially significant mediator or encourager of substance use, especially for adolescents ( Brook *et al.*, 2006), and this could be explored further.

## Data availability

Harvard Dataverse. GAP Year Substance Use Data.

DOI: https://doi.org/10.7910/DVN/YTFKAR

This project contains the following data:

Spreadsheet of socio-demographic, substance use and social support data from the GAP Year trial in two provinces in South Africa;

ACASI (behavioural audio computer-assisted self-interview) Boys and Girls survey codebooks;

Survey and codebook for interviewer-administered questionnaire (2021-10-08)

Data are available under the terms of the Creative Commons Zero "No rights reserved" data waiver (CC0 1.0 Public domain dedication).
