# Supplementary material for: Socio-demographic and social support factors related to substance use in South African in-school adolescents: Insights from the Girls Achieve Power (GAP Year) trial in three peri-urban settings
Source: Gates Open Res. 2021 Nov 9;5:154. [Version 1] doi: 10.12688/gatesopenres.13422.1 (PMC8994034; doi:10.12688/gatesopenres.13422.1)
Supplement: Peer review history

# Peer review: Reviewer response for version 1

**DOI:** 10.21956/gatesopenres.14677.r31858

**Published:** 2022-04-08

**Authors:** Marisa Casale

**Affiliations:** 1 UKRI GCRF Accelerating Achievement for Africa's Adolescents, School of Public Health, University of the Western Cape, Cape Town, South Africa

This is an overall well- and clearly written, well-structured paper focusing on an important topic for public health: prevalence and determinants of substance abuse among South African adolescents. That said, I am not so convinced of the lack of existing research in this area. Substance use is not my specific field of expertise; however a few quick online searches exposed quite a large number of papers focusing on this topic with this population. Granted, some are dated and/or limited in terms of generalizability. However, I do think the authors need to better define the gap and their unique contribution in addressing it. Further more specific comments:

  

- Abstract: on reporting the results the authors should indicate whether these were from an adjusted or unadjusted analysis
- Methods: I think the authors do a good job of describing the processes and procedures. Less so for the measures used and justification for these. Were questions and indicators based on widely used and/or previously validated questions or scales? For example, since social support is a key predictor tested, greater attention should be paid to this construct. The authors should indicate how they define social support, considering that it is a multi-dimensional construct with multiple potential definitions. Also, I note that there are 8 questions assessing social support, but that these are each considered as separate variables in regression analyses. In many cases, social support and its sub-constructs are measured through (validated) scales and subscales, where items are aggregated. It would be useful to know where these questions were taken from, whether they are part of a validated tool, why the items are being considered separately and which dimensions of social support the authors are trying to get to (sources or types of support for example).
- On pg 4 the authors indicate that site selection was done using data to assess the high burden of HIV, GBV and pregnancy. No further detail is provided and it is unclear how this was achieved. The authors should elaborate.
- At various points in the paper the authors rightly highlight the potential strong links between mental health and substance abuse. It is interesting that the authors do not include mental health variables in their analysis (e.g. depression or anxiety) and I wondered whether this was because this data was not available?
- I note the authors indicate that none of the variables remained significant in an adjusted regression analysis for frequency of substance use? Am I correct in understanding that there were therefore no significant predictors once socio-demographic variables were controlled for in the regression? If this is the case, why do the authors feel it is still appropriate to refer to significant predictors in unadjusted analyses in key findings summarized in the abstract? It would seem to me these findings do not hold the same weight as findings in adjusted analyses and could potentially be misleading? If the authors believe findings of unadjusted analyses are important to report, they should go a little further to hypothesise why family support may be an important predictor of substance use, while support from friends may be linked to frequency of use.
- Pg 10: The authors should include citations when referring to the importance of including parents, peers etc in prevention strategies as I believe there are many available resources on this.
- The inability of the authors to use the full number of survey participants because of incomplete questionnaires – and potential bias this may introduce - is an important limitation. I note however that the authors have addressed this in their discussion of limitations.

Is the work clearly and accurately presented and does it cite the current literature?

Yes

If applicable, is the statistical analysis and its interpretation appropriate?

Yes

Are all the source data underlying the results available to ensure full reproducibility?

Yes

Is the study design appropriate and is the work technically sound?

Yes

Are the conclusions drawn adequately supported by the results?

Partly

Are sufficient details of methods and analysis provided to allow replication by others?

Yes

Reviewer Expertise:

Public health; mental health; caregiver and adolescent health; social support; intervention acceptability

I confirm that I have read this submission and believe that I have an appropriate level of expertise to confirm that it is of an acceptable scientific standard.

# Peer review: Reviewer response for version 1

**DOI:** 10.21956/gatesopenres.14677.r31426

**Published:** 2021-12-17

**Authors:** Catherine MacPhail

**Affiliations:** 1 School of Health and Society, Faculty of Arts, Social Sciences and Humanities, University of Wollongong, Wollongong, NSW, Australia

This paper uses the baseline of longitudinal data collected for the GAP project to estimate substance abuse and predictors (of use and frequency) among young South Africans in three communities from the lowest socio-economic strata. The authors note that this information is not routinely available in South Africa, although this seems to be contradicted by the number of citations to South African data that they use, and there is therefore limited evidence from which to derive intervention programmes. The analysis showed a relationship between substance use and age, gender and parental support. Relationships between substance use frequency were also found with age, gender and peer support. The authors note that treatment for substance disorders are limited and therefore research is needed to both advocate for and design such opportunities.

**Abstract:** consider removing ‘A baseline survey gathered demographic and behavioural data’ as this is repetitive of previous text and therefore redundant.

 Would it be possible to slightly alter the language used to discuss the relationship between substance abuse and Family related social support? At the moment the language in the abstract makes it sound as through family support is related with use, while the data shows the opposite.

**Page 3, right column: **replace ‘conducted in 3432 Grade 8 participants’ with ‘conducted with 3432 Grade 8 participants’.

**Page 4, left column:** potentially provide some additional information about the statement ‘site selection was done using data to assess the high burden of HIV, GBV and pregnancy.’ I assume that this is a reference to the outcomes of the GAP evaluation, but it would be useful to clarify this.

 It is unfortunate that the data is not able to link frequency of use to the particular number and type of substance used. Do the authors think that this has implications for the value of the data generated?

**Page 4, right column:** it would be useful for the authors to comment further on the language issue. The survey was translated into isiXhosa for the W Cape participants, but what of the language needs of the participants in Gauteng? The communities sampled would be unlikely to speak English as their first language, although many would be proficient.

 It is interesting that social grant receipt among parents is that low – I would have expected a higher uptake given that low socio-economic communities were particularly targeted in this study. This shows that we need to do more to ensure that eligible individuals are able to access social support!

**Page 10, left column:** I would encourage the authors to unpack the idea about South Africa’s complex relationship with alcohol in more detail. Given that this is an international journal, readers from other contexts may not be aware of the role that alcohol has played in South Africa’s racial history.

**Page 10, right column:** Can the authors expand on the points that they have made about the lack of mental health care options, specifically for adolescents. It would be useful to have some background information here about what service provision levels really are (per 100,000 population for example) and some of the key challenges preventing the South African health care system from providing such care. This would assist in thinking about how to go about ensuring that services are available.

**Page 11, left column:** suggest adding some additional citations to the points being made about social desirability bias and general challenges with administering surveys on sensitive behaviours.

 This paper uses the baseline of longitudinal data collected for the GAP project to estimate substance abuse and predictors (of use and frequency) among young South Africans in three communities from the lowest socio-economic strata. The authors note that this information is not routinely available in South Africa, although this seems to be contradicted by the number of citations to South African data that they use, and there is therefore limited evidence from which to derive intervention programmes. The analysis showed a relationship between substance use and age, gender and parental support. Relationships between substance use frequency were also found with age, gender and peer support. The authors note that treatment for substance disorders are limited and therefore research is needed to both advocate for and design such opportunities.

Is the work clearly and accurately presented and does it cite the current literature?

Yes

If applicable, is the statistical analysis and its interpretation appropriate?

Yes

Are all the source data underlying the results available to ensure full reproducibility?

Yes

Is the study design appropriate and is the work technically sound?

Yes

Are the conclusions drawn adequately supported by the results?

Yes

Are sufficient details of methods and analysis provided to allow replication by others?

Yes

Reviewer Expertise:

HIV prevention, adolescents, domestic violence, social determinants of health

I confirm that I have read this submission and believe that I have an appropriate level of expertise to confirm that it is of an acceptable scientific standard.
